# Expression of Immune Checkpoint Regulators IDO, VISTA, LAG3, and TIM3 in Resected Pancreatic Ductal Adenocarcinoma

**DOI:** 10.3390/cancers13112689

**Published:** 2021-05-29

**Authors:** Felix C. Popp, Ingracia Capino, Joana Bartels, Alexander I. Damanakis, Jiahui Li, Rabi R. Datta, Heike Löser, Yue Zhao, Alexander Quaas, Philipp Lohneis, Christiane J. Bruns, on behalf of the PANCALYZE Study Group

**Affiliations:** 1 Department of General, Visceral, Cancer and Transplantation Surgery, University of Cologne, 50937 Cologne, Germany; felix.popp@uk-koeln.de (F.C.P.); inescapino@gmail.com (I.C.); joanabartels@googlemail.com (J.B.); alexander.damanakis@uk-koeln.de (A.I.D.); jiahuiliss@163.com (J.L.); rabi.datta@uk-koeln.de (R.R.D.); yue.zhao@uk-koeln.de (Y.Z.); 2 Institute of Pathology, University of Cologne, 50937 Cologne, Germany; heike.loeser@uk-koeln.de (H.L.); alexander.quaas@uk-koeln.de (A.Q.); philipp.lohneis@uk-koeln.de (P.L.)

**Keywords:** pancreatic cancer, immune checkpoint, immune molecules, tumor-infiltrating lymphocytes, IDO, VISTA, LAG3, TIM3, Galectin 9, tumor microenvironment

## Abstract

**Simple Summary:**

Pancreatic cancer is deadly, and new treatments are urgently needed. Pancreatic cancer, in particular, effectively escapes the immune response, which is why drugs are developed that stimulate the immune system to remove the tumor. Here, we investigated potential drug targets, the checkpoint inhibitors IDO, VISTA, LAG3, and TIM3. If these checkpoint molecules are associated with poor survival, inhibitory drugs could improve survival. We analyzed 153 pancreatic cancer patients and assessed the expression of checkpoint molecules using immunohistochemistry on tissue microarrays. More than two immune checkpoint molecules were not co-expressed in relevant numbers at the same time. Patients with IDO-expressing tumors had better survival. VISTA, LAG3, and TIM3 expression did not correlate with survival. We expect that immune checkpoint inhibitors against VISTA, LAG3, and TIM3 will not improve patient survival. Our findings complement the picture of pancreatic cancer as highly inaccessible by immune checkpoint inhibitors.

**Abstract:**

Pancreatic cancer features elaborate mechanisms of immune evasion. The potential of new immune molecules was explored to restore the antitumor immune response. If these immune molecules are associated with poor survival, specific drugs could take effect. Here, we analyze the expression of VISTA, LAG3, IDO, and TIM3 on tumor-infiltrating lymphocytes (TILs) and its impact on patient survival. We analyzed 153 pancreatic cancer patients from the prospectively managed database of the multicentered PANCALYZE study. Immunohistochemistry on a tissue microarray assessed VISTA, LAG3, IDO, and TIM3 expression of TILs from the patients undergoing primary resection. Complementarily, we analyzed publicly available transcriptomic data (*n* = 903). Successful completion of chemotherapy, and lymph node status were independent predictors of survival in the multivariate analysis of the clinicopathologic parameters. Fifteen tumors were exclusively VISTA-positive, thirteen tumors expressed VISTA together with TIM3, and ten tumors expressed VISTA together with IDO. Patients featuring tumors with high numbers of IDO-positive TILs had better patient survival (*p* = 0.037). VISTA, LAG3, and TIM3 expression did not correlate with survival. The analysis of publicly available data did not show survival differences. Tumors rarely co-express more than two immune molecules at the same time, and VISTA is most frequently co-expressed. Although IDO generally inhibits T-cell proliferation, a high expression of IDO was associated with improved survival. We expect immune checkpoint inhibitors against VISTA, LAG3, and TIM3 to be inefficient in a clinical application.

## 1. Introduction

The incidence of ductal adenocarcinoma of the pancreas (PDAC) is increasing, and new forms of therapy are urgently needed [1]. For other types of cancer, such as malignant melanoma, immunotherapy has achieved groundbreaking success [2,3,4]. However, pancreatic cancer mostly resists the current immunotherapy, and single-agent regimens have produced only a few responders [5]. The restoration of an effective immune response against pancreatic cancer is still the subject of research [6]. Overcoming pancreatic cancers’ elaborate mechanisms of immune evasion has potential in the development of new therapies [7]. Immune checkpoints are key regulators of immune pathways that suppress or enhance the immune response. Tumors can hijack immune checkpoint molecules to suppress the T-cell response against them. Besides the well analyzed PD-1/PD-L1 axis, novel immune checkpoints like lymphocyte-activation gene 3 (LAG3), T-cell immunoglobulin and mucin-domain containing-3 (TIM3), and V-domain Ig suppressor of T-cell activation (VISTA) have emerged. Other molecules can also influence the immune system and promote tumor growth. Indoleamine 2,3-dioxygenase (IDO), for example, is an intracellular enzyme that produces T-cell-inhibiting metabolites. It catalyzes the rate-limiting step in the catabolism of local tryptophan and therefore contributes to anergy of effector T cells and promotion of regulatory T cells (Tregs) [8]. Various immune cells and stromal cells but also cancer cells express IDO. LAG3 belongs to the immunoglobulin superfamily and is expressed on subsets of B- and T-lymphocytes. LAG3 binds to the major histocompatibility complex 2 (MHC II) to negatively modulate T-cell activity [9]. VISTA, that has some structural similarity to PD-L1, is expressed on tumor-infiltrating lymphocytes (TILs) and myeloid cells and suppresses T-cell activation, proliferation, and cytokine production [10,11]. TIM3 is part of a module containing several checkpoint receptors like PD-1, LAG3, and TIGIT co-expressed on dysfunctional T cells [12]. Co-inhibition of TIM3 enhances the antitumor effect of PD-1 blockade in patients with leiomyosarcoma and non-small-cell lung cancer [12]. Galectin 9 is a ligand of TIM3 that induces the release of BAT3 leading to T-cell inhibition and eventually to cell death [13,14].

The inhibition of T cells through immune checkpoints is observed in various tumors. The main mechanism should also work in pancreatic cancer; possibly, so far, the right immune checkpoints have not been found. Promising immune checkpoints are those for which pharmacological inhibitors already exist. If high immune checkpoint expression is associated with poor survival, these inhibitors could be clinically effective and rapidly adopted as a new therapy. We performed literature research and analyzed ongoing studies. We identified VISTA, LAG3, IDO, and TIM3 as targetable biomarkers and now present a translational study evaluating these immune checkpoints in pancreatic cancer. Recent clinical trials investigate the inhibition of these checkpoints (e.g., LAG-3: NCT02061761; NCT01968109, NCT03538028, NCT03625323, TIM-3: NCT03652077, VISTA: NCT02671955, IDO: NCT01982487, NCT03164603, NCT03695250). Thus, we formulated the hypothesis that elevated numbers of VISTA-, LAG3-, IDO-, and TIM3-positive lymphocytes in the tumor microenvironment of PDAC are associated with poor prognosis. We therefore analyzed the expression of VISTA, LAG3, IDO, and TIM3 in lymphocytes using immunohistochemistry on tissue microarrays (TMAs) of primarily resected ductal adenocarcinomas from the multicentered PANCALYZE trial [15].

## 2. Methods

### 2.1. Patients and Tumor Samples

The reporting recommendations for tumor marker prognostic studies (REMARK) were followed for reporting this study [16]. The PANCALYZE study cohort was used for this analysis and provided the baseline data and patient characteristics. Between 2014 and 2018, a total of 153 patients with primary resected PDAC enrolled in this multicenter study. Surgery was performed according to the German clinical guideline for PDAC without specific requirements from the study protocol. Formalin-fixed, paraffin-embedded (FFPE) tumor samples obtained after resection were sent to the University Hospital of Cologne to establish a central biobank. Follow-up assessed recurrence pattern, survival time, and adjuvant tumor therapy. It was performed by telephone interviews with the attending oncologist at the coordinating center. The study was approved by the institutional review committee and the responsible ethics committees of the participating centers (registration number DRKS00006179; German Clinical Trials Register—DRKS; www.germanctr.de, accessed on 27 May 2021). Staging of tumors was according to UICC guidelines.

TMAs were constructed using tumor cylinders, with a diameter of 1.2 mm, that were punched out of the tissue samples using a self-constructed, semi-automatic precision instrument. The punches were embedded in empty recipient paraffin blocks. Sections, 4 μm thick, of the resulting TMA blocks were transferred to an adhesive-coated slide system (Instrumedics Inc., Hackensack, NJ, USA) for immunohistochemistry.

### 2.2. Immunohistochemistry

Immunohistochemical staining was performed according to standard procedures on a Leica Bond Max™ system (Leica Microsystems, Wetzlar, Germany) using a monoclonal antibody directed against VISTA (D1L2G; 1:100; Cell Signaling Technology, Leiden, The Netherlands), IDO (D5J4E; 1:400; Cell Signaling Technology), TIM3 (D5D5R; 1:100; Cell Signaling Technology), LAG3 (D2G40; 1:300; Cell Signaling Technology,), and Galectin 9 (D9R4A; 1:200; Cell Signaling Technology).

Tumor infiltration with CD3 cells was evaluated semiquantitatively. Less than 3 cells per mm^2^ were defined as no infiltration (score 1), detection of 3–50 cells/mm^2^ was defined as low infiltration (score 2), and detection of more than 50 cells/mm^2^ was regarded as high infiltration (score 3).

The expression of VISTA, IDO, LAG3, TIM3, and Galectin 9 on inflammatory cells was semiquantitatively assessed as previously published [17,18,19]. Briefly, expression in less than 1% of TILs was defined as negative (score 0), in 1–4% was assessed as low expression (score 1), >4% was regarded as high expression (score 2). For the analysis, we chose a cutoff of 2 because we expected a biological effect only of strong-positive-expressing immune checkpoints. At a score of 1, only up to 5% of tumor-infiltrating lymphocytes are positive, so we combined the scores 1 and 0 to the expectedly non-biologically active (negative) group. 

For the LAG3 analysis, we decided to use a cutoff of 1, firstly because there are already publications using this cutoff and, secondly, only one tumor had a LAG3 score of 2.

The proportion of positive inflammatory cells was determined on immunostained TMA slides by an experienced pathologist (PL) blinded to clinical outcome. Results were checked for consistency by a second investigator (HL). Discrepant results were resolved by consensus review.

### 2.3. Analysis of Publicly Available Transcriptomic Data

We used curated pancreatic cancer patient data from the MetaGxData project. Gendoo et al. pooled 11 datasets of publicly available RNA sequencing data and annotated standardized clinical, pathological, and survival data [20]. For the analysis, we downloaded the MetaGxData project programmed in R from CodeOcean (https://codeocean.com/capsule/6438633/, accessed on 30 April 2021) and installed the necessary libraries from Bioconductor’s ExperimentHub (https://bioconductor.org/, accessed on 30 April 2021) [21,22]. We modified the R script to calculate the survival of PDAC patients with available expression data for IDO, VISTA, LAG3, TIM3, and Galectin 9 (see supplemental file). To make the survival analysis comparable with the TMA analysis, we divided the patients from each of the 11 datasets into two groups using the 66th percentile. We combined the survival data from each dataset to calculate the total survival of the 11 datasets.

### 2.4. Statistical Analysis

Disease-free survival (DSF) was defined as the time from surgery to local or distant disease relapse and overall survival (OS) was defined as the time from surgery to death of any cause. The Kaplan–Meier method with log-rank tests was used for univariate survival analyses. Calculating Schoenfeld residuals revealed that the proportional hazards assumption for the multivariate cox regression analysis was violated. To account for time-dependent effects of covariates, we used weighted Cox regression for multivariate analysis [23,24]. Pearson’s correlation method was used to correlate expression of immune checkpoints and clinicopathological parameters. In general, two-sided *p* values were calculated and considered to be significant when <0.05. The software R [25], RStudio (RStudio PBC, Boston, MA, USA) [26], GraphPad Prism (version 7; GraphPad Software, Inc., San Diego, CA, USA), and Microsoft Excel (Microsoft Corp., Redmond, WA, USA) helped to perform the statistical analysis and to generate the figures.

## 3. Results

### 3.1. PANCALYZE Study Cohort and Clinicopathologic Parameters

The multicenter PANCALYZE study cohort consisted of 153 patients that all had a known survival status. The median age of patients was 69.7 years (range 46–89 years). There were and 72 (47.1%) women and 81 (52.9%) men. The median follow-up time was 2.2 years. During the observation period, we found no evidence of disease in 27 (17.6%) patients. A total of 35 (22.9%) patients were alive with recurrent pancreatic cancer. Of the 153 patients, 81 (52.9%) died during the follow-up. Of the 81 deceased patients, 53 (34.6%) had recurrent disease, 36 (23.5%) patients had no recurrence. For two patients (1.3%), the recurrence status could not be assessed. The cause of death was not explicitly surveyed. During the follow-up, we assessed the location of the first recurrence. Most patients developed liver metastasis (30%), peritoneal carcinomatosis (28%), or local recurrence (25%) as the first recurrence (see Table 1). Median survival was 1.2 years, and median DSF was 0.8 years for all patients. Patients completing the adjuvant chemotherapy had a median survival of 1.8 (0.5–4.5) years. 

**Figure 1:**
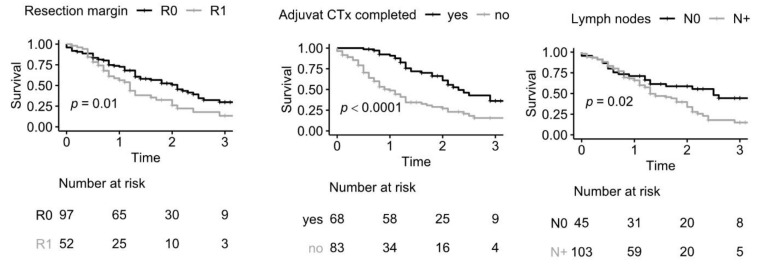

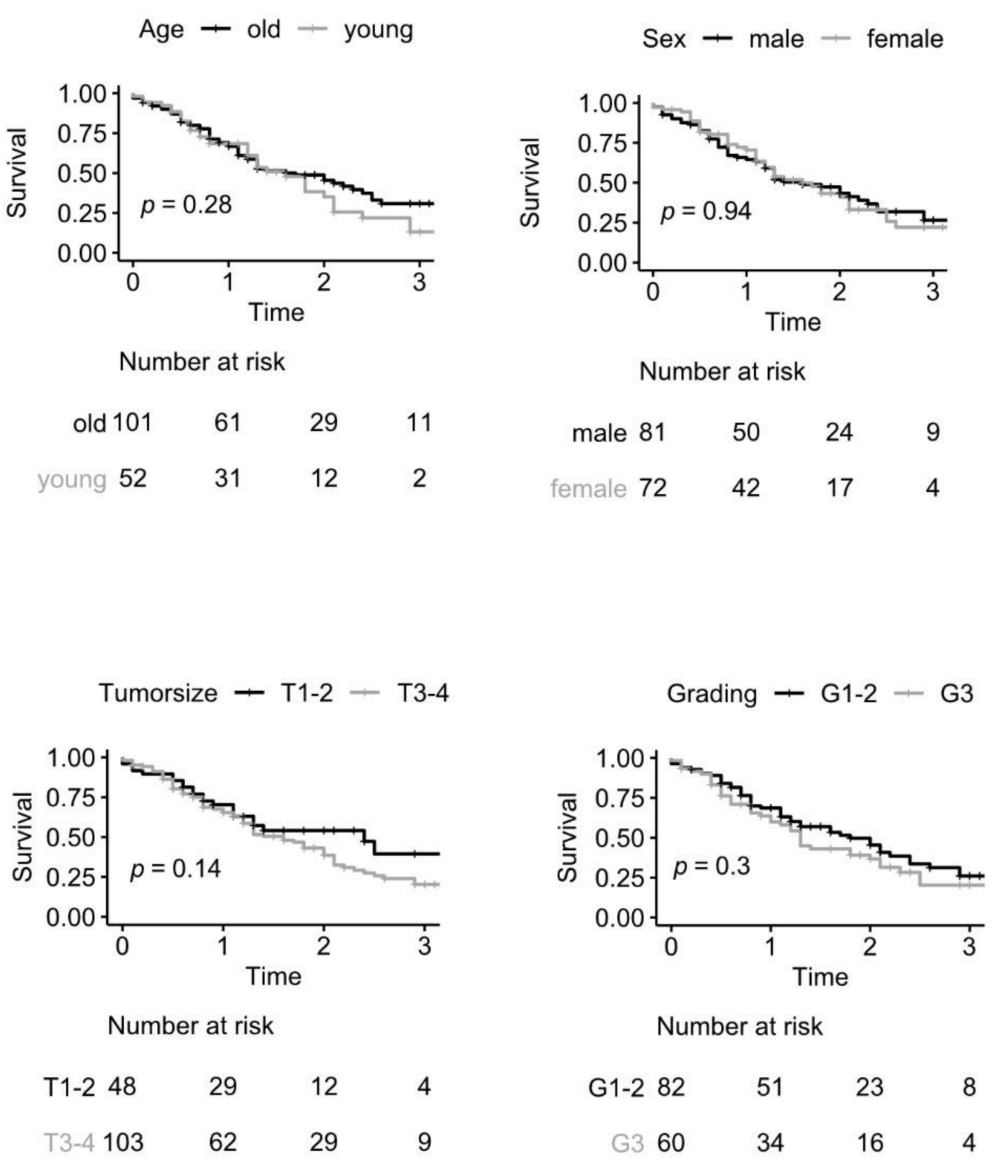
Kaplan–Meier estimates of overall survival stratified by resection margin (R0 vs. R1, *p* = 0.0079), successful completion of adjuvant chemotherapy (*p* < 0.0001), lymph nodes (N0 vs. N+, *p* = 0.02), age (old (≥65 years) vs. young (<65 years), *p* = 0.28), sex (female vs. male, *p* = 0.94), tumor size (T1–2 vs. T3–4, *p* = 0.14), and grading (G1–2 vs. G3, *p* = 0.3).

In the univariate Cox regression analysis, the resection margin (R0 vs. R1), the lymph node status (N0 vs. N+), and successful completion of adjuvant chemotherapy were statistically significant. The discrimination between old (≥65 years) vs. young (<65 years), female vs. male patients, T1–2 vs. T3–4 tumors, and G1–2 vs. G3 tumors showed no significant difference in the risk of death (see Table 2). The corresponding survival curves are shown in Figure 1.

In the multivariate analysis of the clinicopathologic parameters, the successful completion of chemotherapy, and the lymph node status were independent predictors of survival. The resection margin, age, sex, tumor size (T-stage), and degree of differentiation (grading) were not independent predictors of cancer-specific survival (see Table 3). 

### 3.2. Expression of the Immune Checkpoints VISTA, IDO, LAG3, and TIM3 in Pancreatic Ductal Adenocarcinoma

**Figure 2:**
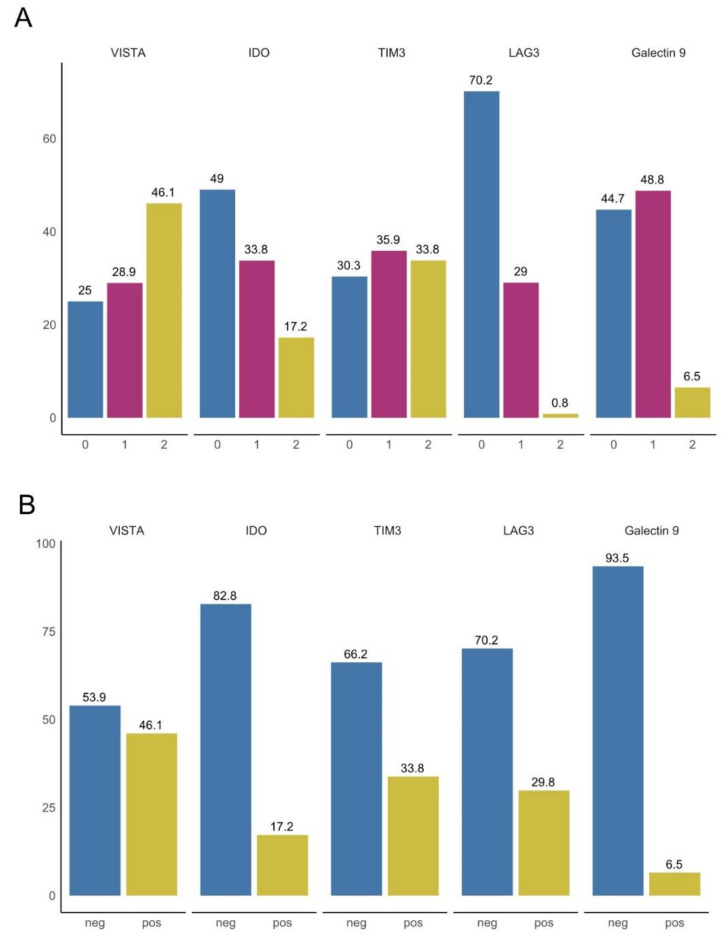
Expression of checkpoint molecules in the pancreatic cancer cohort. (**A**) For VISTA, 38 (25.0%) tumors were negative (0), while low (1) and high (2) expression was detected in 44 (28.9%) and 70 (46.1%) tumors. IDO was negative in 74 (49%) tumors, low and high expression was detected in respectively 51 (33.8%) and 26 (17.2%) tumors. TIM3 was negative in 43 (30.1%) tumors, low and high expression was detected in respectively 52 (36.4%) and 48 (33.6%) tumors. LAG3 was negative in 86 (69.9%) tumors, low and high expression was detected in respectively 36 (29.3%) and 1 (0.8%) tumor. (**B**) Negative and low-expressing tumors were combined into one group (neg) and compared with high-expressing tumors (pos) for the analysis of VISTA, IDO, and TIM3. Because there was only one LAG3-positive-expressing tumor, we combined the positive tumor with the low-expressing tumors into the positive group (pos) for LAG3 analysis.

Expression of the immune checkpoints VISTA, IDO, TIM3, and LAG3 was evaluable in respectively 152, 153, 145, and 124 tumors. Of all TILs, 46.1% were VISTA-positive-expressing (>4%), 28.9% low-expressing (1–4%), and 25% negative (<1%). We detected IDO-positive-expressing TILs in 17.2%, low-expressing TILs in 33.8%, and negative TILs in 49.0% of all tumors. The distribution of the TIM3 expression was homogeneous. We observed 33.8% positive-expressing, 35.9% low-expressing, and 30.3% negative TILs. There was only one LAG3-positive tumor (0.8%) with more than 4% LAG3-positive TILs, 29.0% of tumors had low-LAG3-expressing TILs, and 70.2% of tumors showed no LAG3-positive TILs (see Figure 2A). For the analysis of VISTA, IDO, and TIM3, we combined the negative and low-expressing tumors into one group (negative group). Because there was only one LAG3-positive-expressing tumor, we combined the positive tumor with the low-expressing tumors into the positive group for LAG3 analysis (see Figure 2B).

### 3.3. Correlation of VISTA, IDO, LAG3, and TIM3 Expression with Clinicopathological Parameters

We assessed the correlation of clinicopathological parameters with the expression of the immune checkpoints VISTA, IDO, LAG3, and TIM3 in the tumors (Table 4). 

We found no dependencies between patients age (<65 and ≥65 years), sex (male and female), pT classification (pT1/2 and pT3/4), nodal status (pN0 and pN+), resection margins (R0 and R1), grading (G1/2 and G3), and pattern of recurrence and the expression of the immune checkpoints, except for a correlation between TIM3 expression and the T status.

### 3.4. High Expression of IDO but Not VISTA, LAG3, and TIM3 Is Associated with Improved Survival in Resected Pancreatic Ductal Adenocarcinoma

**Figure 3:**
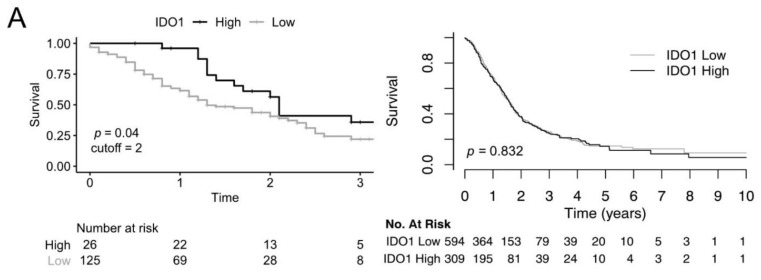

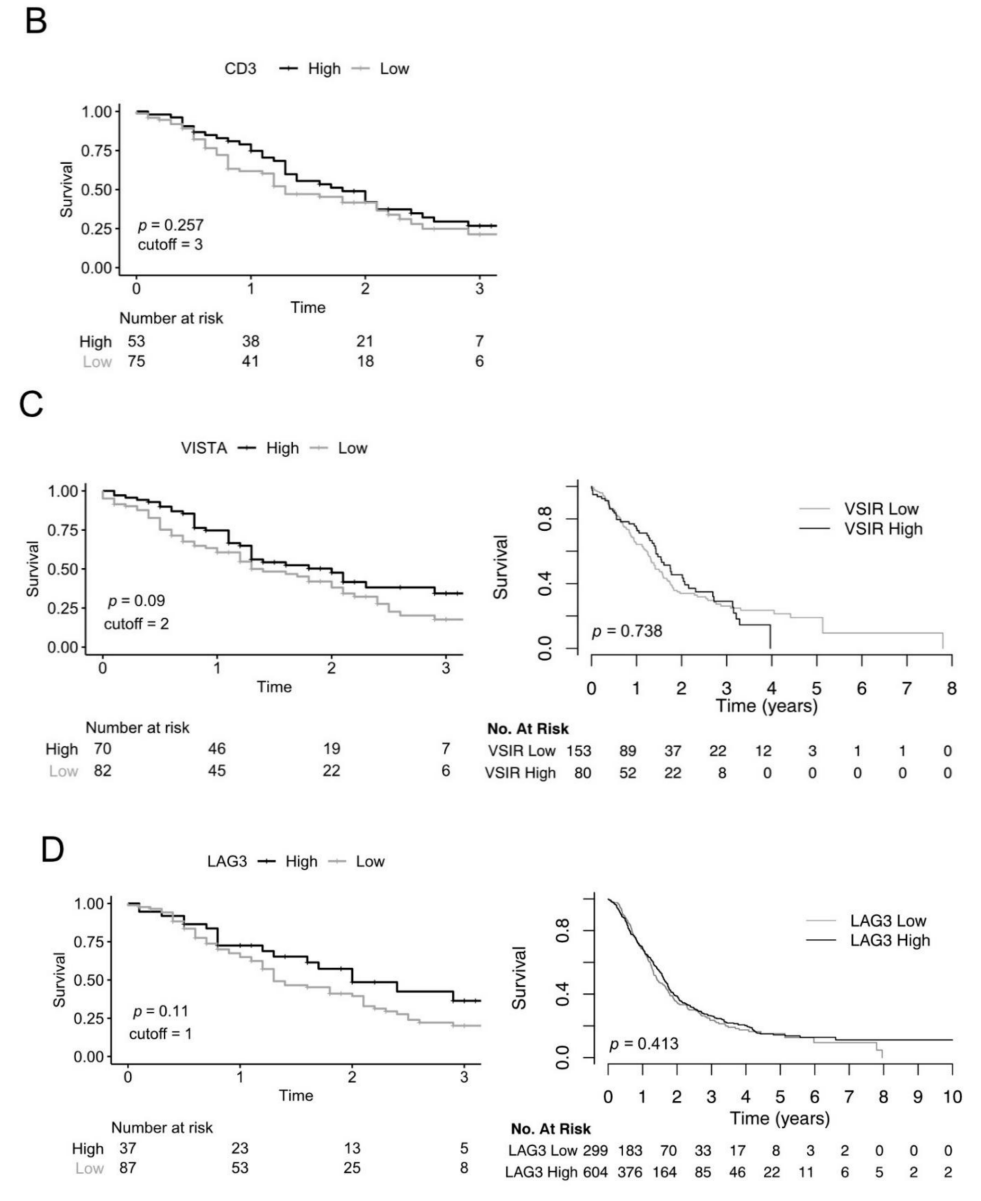

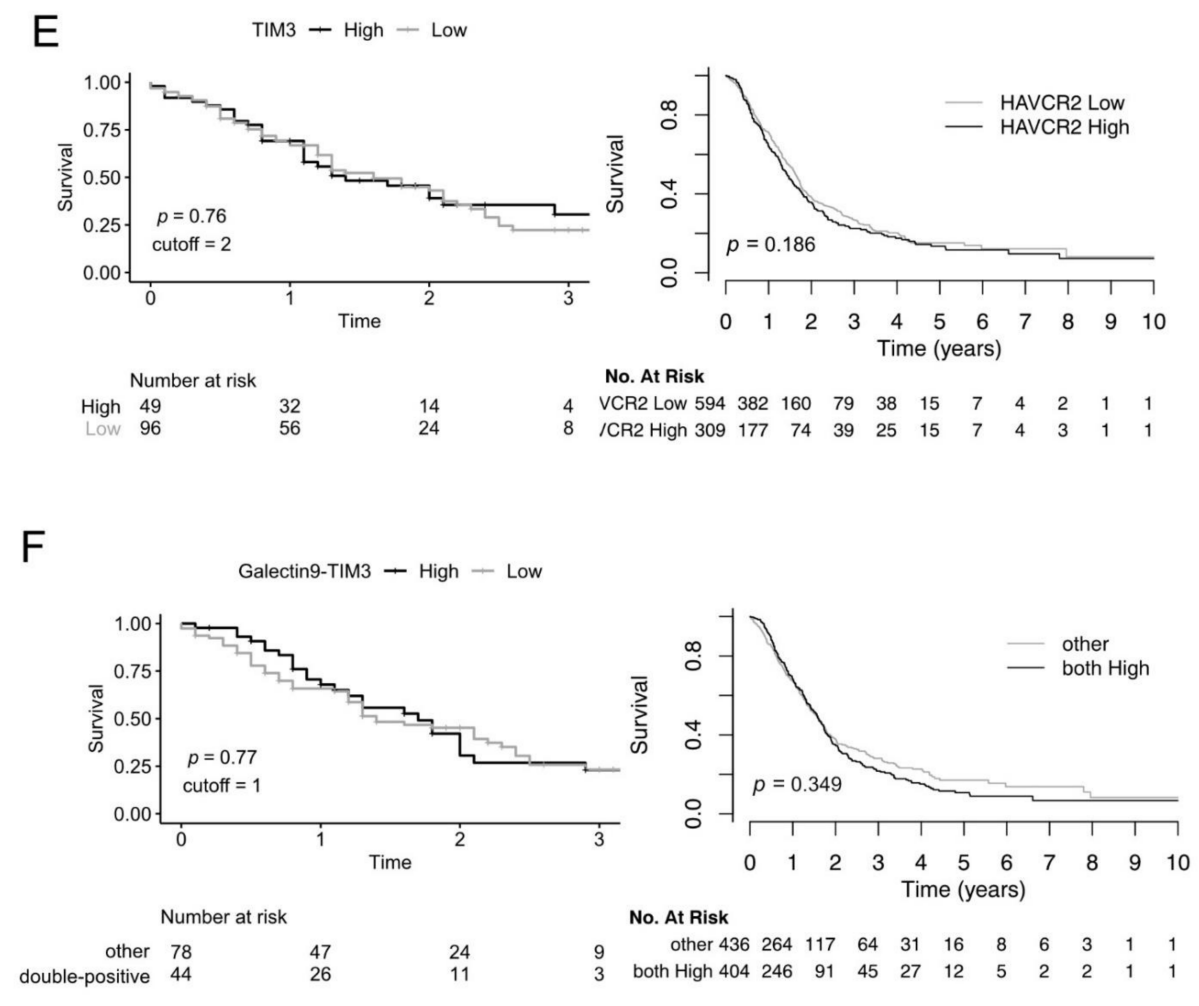
Overall survival related to the expression of the immune molecules. (**A**) Kaplan–Meier estimates of overall survival of patients with IDO-positive tumors (high) vs. IDO-negative/low-expressing tumors (low) assessed with immunochemistry on the TMA (left, *n* = 151, *p* = 0.037, log-rank test). Survival of patients from public transcriptomic databases with IDO-low-expressing (≤66th percentile) vs. IDO-high-expressing (>66th percentile) tumors. The overall survival is statistically equivalent (*n* = 903, *p* = 0.832, log-rank test, right). (**B**) Survival of patients with CD3-high infiltrating tumors (high - score 3) vs. CD3-low infiltrating tumors (low - scores 1 and 2; *n* = 128, *p* = 0.257, log-rank test). (**C**) Survival of the TMA cohort patients with VISTA-positive tumors (high) vs. VISTA negative/low-expressing tumors (low) (left, *n* = 152, *p* = 0.091, log-rank test). Survival of patients from public transcriptomic databases with VSIR-low-expressing (≤66th percentile) vs. VSIR-high-expressing (>66th percentile) tumors. The overall survival is statistically equivalent (*n* = 233, *p* = 0.738, log-rank test, right). VSIR encodes the VISTA protein. (**D**) Survival of the TMA cohort patients with LAG3-positive/low-expressing tumors (positive) vs. LAG3 negative-expressing tumors (negative) (left, *n* = 124, *p* = 0.11, log-rank test). Survival of patients from public transcriptomic databases with LAG3-low-expressing (≤33rd percentile) vs. LAG3-high-expressing (>33rd percentile) tumors. The overall survival is statistically equivalent (*n* = 903, *p* = 0.413, log-rank test, right). (**E**) Survival of the TMA cohort patients with TIM3-positive tumors (high) vs. TIM3-negative/low-expressing tumors (low) (left, *n* = 145, *p* = 0.76, log-rank test). Survival of patients from public transcriptomic databases with HAVCR2-low-expressing (≤66th percentile) vs. HAVCR2-high-expressing (>66th percentile) tumors. The overall survival is statistically equivalent (*n* = 903, *p* = 0.186, log-rank test, right). HAVCR2 encodes the TIM3 protein. (**F**) Survival of Galectin 9–TIM3 double-positive patients (double-positive) vs. the remaining patients (other, left, *n* = 122, *p* = 0.77, log-rank test). Survival Galectin 9–TIM3 high (both high) patients from public transcriptomic databases (>33rd percentile) vs. Galectin 9–TIM3-low-expressing (≤33rd percentile) tumors. The overall survival is statistically equivalent (*n* = 208, *p* = 0.349, log-rank test, right).

When correlating the expression of IDO, VISTA, LAG3, and TIM3 with survival, we unexpectedly found IDO-positive tumors associated with a significantly longer overall survival (OS) in patients with resected pancreatic adenocarcinomas. Median OS/DFS were 1.3/1 years for IDO-negative/low-expressing tumors and 2.1/1.3 years for IDO-positive tumors (*p* = 0.037/0.14, HR 1.8/1.5; see Figure 3A). IDO (2 vs. 0–1) expression and the nodal status (pN0 and pN+) were independent predictors of cancer-specific survival in the multivariate analysis, but sex (male and female), age (<65 and ≥65 years), and grading (G1/2 and G3) were not (see Table 5). To check whether IDO expression correlates with infiltration of CD3-positive immune cells, we evaluated CD3 infiltration semiquantitatively. Survival with high CD3 infiltration was not longer compared with low infiltration (*n* = 128, *p* = 0.257; see Figure 3B). Calculation of the Spearman correlation of CD3 and IDO yielded a correlation coefficient of rho = 0.29. This correlation was significant in the one-tailed correlation test (*p* < 0.001). Together, this indicates a weak correlation, which the contingency table also shows (see Table 6). To complement our survival analysis, we analyzed publicly available transcriptomic data. We used the MetaGxPancreas dataset, which comprises RNA sequencing data from multiple sources as well as manually curated clinical and survival data. IDO expression was available for 903 patients. Dividing the dataset into IDO-low- and IDO-high-expressing tumors (≤66th percentile and >66th percentile, respectively) did not result in different overall survival (*p* = 0.832; see Figure 3A).

VISTA-positive tumors were not associated with a longer OS in patients with resected pancreatic adenocarcinomas. Median OS/DFS was 1.3/1.1 years for VISTA-negative/low-expressing tumors and 1.8/1 years for VISTA-positive tumors (*p* = 0.1/0.41, HR 1.4/1.2; see Figure 3C). The publicly available transcriptomic data confirmed this result. Dividing the dataset at the 66th percentile produced no survival difference (*p* = 0.738; see Figure 3C).

There was only one LAG3-positive tumor. For the survival analysis, we combined the positive (score 2) and low-positive (score 1) tumors. For LAG3, median OS/DFS were 1.3/1.1 years for negative tumors and 2.0/1.1 years for low/positive-expressing tumors (*p* = 0.11/0.89, HR 1.5/1.0; see Figure 3D). Following the grouping of the TMA data, we split the publicly available transcriptomic data at the 33rd percentile. There was no survival difference (*p* = 0.413; see Figure 3D). 

**Figure 4:**
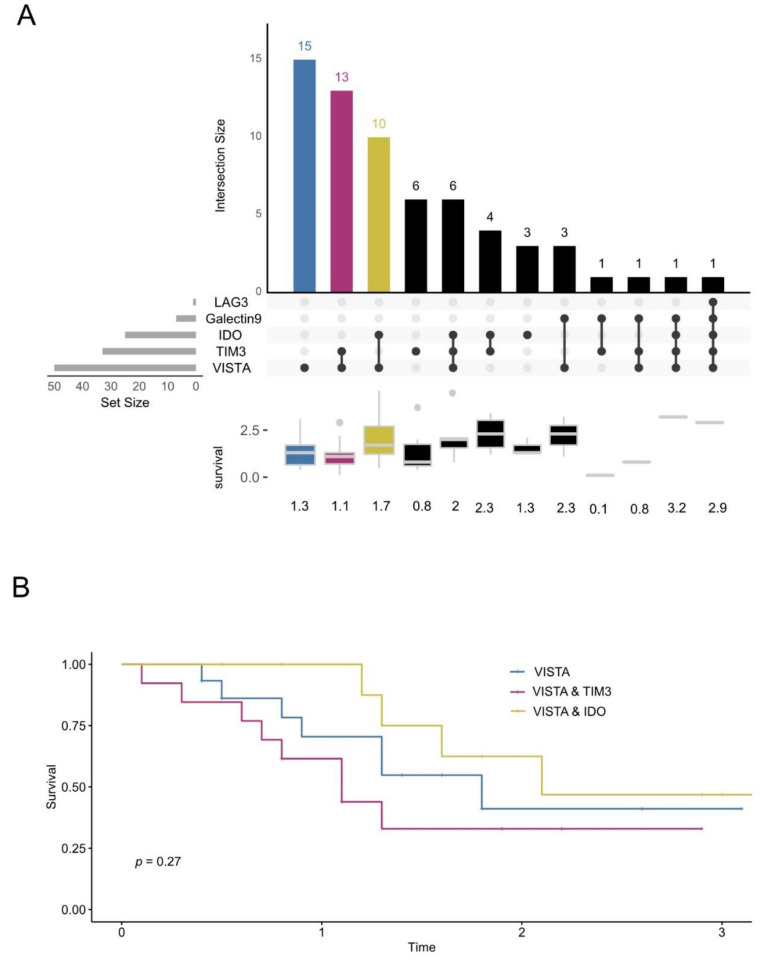
UpSet plot to visualize the intersecting sets of immune molecule expression. (**A**) VISTA was the most frequently expressed immune molecule (44% of all tumors, *n* = 113), Fifteen tumors expressed VISTA alone (13%) and no other immune molecules, followed by thirteen tumors expressing VISTA and TIM3 only (12%), and ten tumors expressing VISTA and IDO (9%). The box plot below depicts the median survival of the immune molecule combinations depicted in the UpSet plot above. (**B**) Survival of the patients with VISTA-, VISTA- and TIM3-, and VISTA- and IDO-expressing tumors (*n* = 38, *p* = 0.27, log-rank test).

TIM3-positive tumors were not associated with a longer OS. Median OS/DFS were 1.6/1.0 years for TIM3-negative/low-expressing tumors and 1.4/0.9 years for TIM3-positive tumors (*p* = 0.81/0.73, HR 1.1/1.1; see Figure 4A). The publicly available transcriptomic data strengthened this result. Dividing the dataset at the 66th percentile produced no survival difference (*p* = 0.186; see Figure 4B). 

Galectin 9 is a ligand of TIM3. Possibly both checkpoint molecules need to be expressed for TIM3 to have an immunomodulatory effect. Therefore, we assessed the expression of Galectin 9 by immunohistochemistry. Only 6.5% of the tumors were Galectin 9-positive, but roughly half of the tumors were low-positive (48.8%; see Figure 2). For the survival analysis, we combined the positive (score 2) and low-positive (score 1) tumors for both markers Galectin 9 and TIM3. Thus, we defined double-positive tumors as any combination not containing negative tumors (score 0) of Galectin 9 and TIM3 expression (see Table 7). However, neither the immunohistochemical analysis (*n* = 122, *p* = 0.77, see Figure 3F) nor the publicly available transcriptomic data split at the 33rd percentile (for Galectin 9 and TIM3) showed differences in survival of patients with double-positive tumors (*p* = 0.35; see Figure 3F). 

To better understand the co-expression pattern of checkpoint molecules, we studied expression sets and their intersections [27]. We could determine the expression of all five checkpoint molecules completely in 113 tumors. Most frequently, the tumors expressed VISTA (*n* = 50). Of these, fifteen tumors were exclusively VISTA-positive and did not express any other checkpoint molecules. Thirteen tumors expressed VISTA together with TIM3, and ten tumors expressed VISTA together with IDO. Six tumors expressed TIM3 alone, and three tumors expressed IDO exclusively (see Figure 4A). The median survival of the three largest groups did not differ significantly (VISTA, VISTA and TIM3, VISTA and IDO, *p* = 0.27; see Figure 4B).

## 4. Discussion

Immune cells in the tumor microenvironment (TME) regulate the initiation and progression of PDAC. They establish the tolerogenic niche in PDAC. The distribution of immune cells in pancreatic cancer is highly variable [28,29,30] and influences therapy outcome. Several studies could show that enrichment of CD8-positive effector T cells in the tumor is associated with a favorable prognosis in PDACs [31,32]. Immune checkpoint proteins are inhibitory or stimulatory co-signaling molecules on the surface of immune effector cells. Inhibitory co-signaling leads to T cell exhaustion and immune evasion. The pharmacological inhibition of immune checkpoints could therefore restore an antitumor immune reaction. Thus, immunotherapy has established itself as a mainstay in the treatment of many tumors [33]. A corresponding success in pancreatic cancer has so far failed to materialize [34]. In a phase 1 trial with an anti-PD-L1 blocking antibody, no objective response was observed in PDAC patients [35]. Blocking PD1 is effective in tumors with microsatellite instability because the underlying DNA mismatch repair defects induce high numbers of mutations and neoantigens [36]. However, the vast majority of PDAC tumors do not exhibit microsatellite instability. To tackle immune evasion by pancreatic cancers, the combination of anti-PD-L1 and anti-CTLA-4 checkpoint inhibitors was investigated in patients with metastatic disease. Again, only 3% of patients responded [37]. Other attempts such as vaccination therapy [38], targeting of myeloid cells [39], and CAR-T cell therapy [40] showed promise in animal and phase 1 studies. In clinical trials, these immunotherapies have not yet met expectations [41,42]. Thus, successful immunotherapy must approach all mechanisms of immune invasion. Basic science has shown that pancreatic cancer has multiple immune defects that prevent successful immunotherapy [43]. These include a heterogeneous dense stroma forming a physical barrier and an immunosuppressive tumor microenvironment. Intratumoral effector T cells are dysfunctional and fail to eliminate tumors. To develop novel multicombination therapies, we investigated the potential immune checkpoint markers VISTA, LAG3, IDO, and TIM3 in patients of the PANCALYZE study. 

The multicenter PANCALYZE study cohort is a representative cross-section of German pancreatic cancer patients. The population of 153 analyzed patients is homogenous because only patients undergoing routine surgery for pancreatic cancer registered for this study [15]. The median survival of 1.8 years after completion of adjuvant chemotherapy is typical for these PDAC patients [44].

We analyzed the co-expression pattern of VISTA, LAG3, IDO, and TIM3 on TILs. Interestingly, the tumors rarely co-express more than two immune molecules simultaneously (see Figure 4). Tumors express VISTA most often, either alone or together with TIM3 and IDO. We expected a more balanced distribution pattern. The combined expression of multiple immune molecules did not change survival.

Unexpectedly, tumors with higher numbers of IDO-positive TILs correlated with better patient survival. IDO deprives T cells of tryptophan, which leads to a decreased T cell response and T cell anergy [45,46]. Consequently, the attenuated immune response promotes tumor growth, and patients with IDO-positive tumors should survive for a shorter time. IDO expression correlates with poor prognosis in advanced gastric cancer in one study [47], and data from basic science clearly point to a tumor-promoting effect of IDO [8,48]. However, elevated IDO expression has been associated with an improved survival of gastric carcinomas [49], basal-like breast cancer [50], cervical cancer [51], renal cell carcinomas [52], and esophageal adenocarcinomas [17]. In pancreatic carcinomas, Sideras et al. published a survival advantage for patients with IDO-positive tumors, suggesting that our observation is not a random event [53]. In the PANCALYZE cohort, infiltration with CD3-positive cells was not associated with better survival. Tumor infiltration with CD3-positive cells correlated only weakly with IDO expression. Thus, IDO expression is not a marker of enhanced immune infiltration. We controlled the result from our TMA analysis using publicly available transcriptomic data and found no survival advantage for patients with IDO-positive tumors in this large patient group (*n* = 903). We analyzed IDO expression selectively on TILs. IDO transcriptome analysis, in contrast, analyzes the IDO expression of all cells in the tumor. Thus, the additionally captured IDO expression of tumor cells may explain the diverging findings. Basic science results highlight the immunosuppressive role of IDO in the lymph node [45]. It could be that IDO production in the tumor plays a minor role compared to expression in the lymph node. 

Sideras et al. published encouraging results regarding Galectin 9 expression [53]. In contrast, we did not find a correlation between Galectin 9 expression and survival (see supplementary Figure S1). For TIM3, the ligand of Galectin 9, we found no such correlation either. In double-positive tumors, the ligands should be able to bind to each other and exert their immunomodulatory effect. Even when only looking at TIM3/Galectin 9 double-positive tumors, there is no correlation between checkpoint expression and survival. All analyses were double-checked using the publicly available transcriptomic data and produced the same results as the TMA analysis. The other checkpoint molecules VISTA and LAG3 also showed no correlation to survival. However, there were only very few LAG3-positive tumors. In contrast to our own results, a recent study described a reduced disease-free survival of patients with PDAC with LAG3-expressing T cells on a smaller cohort of PDAC [54]. When analyzing the immune checkpoints IDO, TIM3, and VISTA with a cutoff of 1 or with respect to the individual scores, we found no survival differences. In these examinations, negative tumors had a score of 0, and positive tumors had a score of 1 or 2 (see supplementary Figure S2).

One would expect that checkpoint inhibitors can improve survival if the corresponding checkpoint molecules are associated with poor survival. Our data show no survival difference for VISTA-, LAG3-, and TIM3-positive tumors. These findings join the list of low-impact immune checkpoints and complement the picture of pancreatic cancer as an extremely immune-evasive tumor. We expect that immune checkpoint inhibitors against VISTA, LAG3, and TIM3 will also not affect patient survival. 

These results raise the question of the importance of the distribution of immune checkpoint molecules in the tumor itself. The tumor’s footprint in the immune system could determine survival. Tumors might change the immune system not only locally. After tumor removal, the systemic immune changes could define recurrence and thus influence survival much more than the local microenvironment. TMA analysis detects only the immediate microenvironment of the tumor. Future analysis of lymph nodes could yield a systemic expression pattern of immune checkpoint molecules. 

## 5. Conclusions

Tumors co-express VISTA most frequently but with few other immune checkpoints. More than two immune checkpoint molecules are not co-expressed in relevant numbers at the same time. High IDO expression was associated with better survival. Several other studies confirmed our finding in pancreatic cancer and other tumor entities. We do not believe that IDO is only a marker for immune infiltration. IDO’s survival benefit is most likely an independent effect and warrants further research.

Our findings complement the picture of pancreatic cancer as highly inaccessible to immune checkpoint inhibitors. We expect that immune checkpoint inhibitors against VISTA, LAG3, and TIM3 will not improve patient survival. The unique tissue microenvironment of pancreatic cancer prevents effective immunotherapy. However, combining immunotherapy with other therapies like microRNA targeting angiogenesis [55] might restore the antitumor immune response. Anti-angiogenic therapy possibly remodels the tissue microenvironment to become more accessible for immunotherapy. Following that route, it may still be possible to establish effective immune checkpoint therapy after all—if not combining multiple checkpoint inhibitors, then along with synergistic therapies targeting other components of the TME.

## Figures and Tables

**Table 1:** Baseline characteristics, histopathological findings, and pattern of first recurrence of the patients of the PANCALYZE study cohort.

Characteristic	N = 153
Sex	
Male	81 (53%)
Female	72 (47%)
Age	
Old	101 (66%)
Young	52 (34%)
Resection margin	
R0	97 (65%)
R1	52 (35%)
R2	1 (0.7%)
RX	3
Tumor size	
T1	11 (7.3%)
T2	37 (25%)
T3	97 (64%)
T4	6 (4.0%)
Unknown	2
Lymph nodes	
N0	45 (30%)
N1	52 (35%)
N2	51 (34%)
Unknown	5
Grading	
G1	2 (1.4%)
G2	80 (56%)
G3	60 (42%)
Unknown	11
Adjuvant CTx completed	
Yes	68 (45%)
No	83 (55%)
Unknown	2
Site of first recurrence	
Liver metastasis	28 (30%)
Local recurrence	23 (25%)
Lung metastasis	10 (11%)
Peritoneal carcinomatosis	26 (28%)
Other type of recurrence	6 (6.5%)
Unknown	60
*n* (%)	

**Table 2:** Univariate Cox proportional hazard regression analysis of patient survival.

Groups	HR	95% CI	*p*-Value
R0 vs. R1	1.772	1.16–2.72	0.0087
completed adjuvant chemotherapy yes vs. no	2.782	1.79–4.32	<0.00001
old (≥65) vs. young (<65 yrs.)	1.268	0.83–1.94	0.276
female vs. male	1.018	0.67–1.54	0.934
N0 vs. N+	1.817	1.11–2.96	0.017
T1–2 vs. T3–4	1.433	0.89–2.32	0.143
G1–2 vs. G3	1.249	0.82–1.90	0.3

**Table 3:** Multivariate Cox proportional hazard regression analysis of clinicopathological factors influencing survival after pancreatic cancer resection. The successful completion of chemotherapy, and the lymph node status were independent predictors of survival.

Characteristic	HR	95% CI	*p*-Value
Age			0.55
Old	—	—	
Young	1.15	0.73, 1.83	
Sex			0.68
Male	—	—	
Female	1.10	0.7, 1.73	
Tumor size			0.44
T1–2	—	—	
T3–4	1.26	0.7, 2.26	
Lymph nodes			0.022
N0	—	—	
N+	2.0	1.11, 3.66	
Resection margin			0.3
R0	—	—	
R1	1.3	0.79, 2.15	
Grading			0.234
G1–2	—	—	
G3	1.32	0.84, 2.07	
Adjuvant CTx completed			<0.001
Yes	—	—	
No	3.14	1.99, 4.95	

HR = hazard ratio, CI = confidence interval.

**Table 4:** Correlation of clinicopathological parameters with the expression of the immune checkpoint molecules IDO, VISTA, TIM3, and LAG3. The chi-square test for independence was used to analyze the correlation table formed by two categorical variables at a time.

Chi-Squared *p*	IDO	VISTA	TIM3	LAG3
Age	0.3294	0.6983	0.4987	0.3424
Sex	0.226	0.676	0.495	0.2114
T status	0.6721	0.2965	0.04396	0.5433
N status	0.3391	0.6098	0.2221	0.9885
R status	0.7408	0.2169	0.1942	0.34
Grading	0.571	0.5704	0.2533	0.08138
Recurrence	0.7008	0.6908	0.7635	0.6899

**Table 5:** Multivariate Cox proportional hazard regression analysis for IDO expression and clinicopathological factors. IDO expression and lymph node status are independent predictors for survival.

Characteristic	HR	95% CI	*p*-Value
IDO Expression			0.009
High	—	—	
Low	2.09	1.2, 3.62	
Sex			0.654
Male	—	—	
Female	1.11	0.71, 1.72	
Age			0.353
Old	—	—	
Young	1.24	0.79, 1.95	
Lymph nodes			0.033
N0	—	—	
N+	1.9	1.05, 3.44	
Grading			0.268
G1–2	—	—	
G3	1.28	0.83, 1.99	

HR = hazard ratio, CI = confidence interval.

**Table 6:** Contingency table of CD3 versus IDO.

No. of Cases	IDO			
(frequency)	0	1	2	Total
CD3				
1	3 (2.4%)	0 (0%)	0 (0%)	3 (2.4%)
2	42 (33%)	19 (15%)	10 (7.9%)	71 (56%)
3	18 (14%)	19 (15%)	16 (13%)	53 (42%)
Total	63 (50%)	38 (30%)	26 (20%)	127 (100%)

**Table 7:** Definition of Galectin 9/TIM3 double-positive tumors as any combination not containing negative tumors (score 0).

Galectin 9 Score	TIM3 Score	Group
0	0	other
0	1	other
1	0	other
1	1	double-positive
1	2	double-positive
2	1	double-positive
2	2	double-positive

## Data Availability

The data that support the findings of this study are available from the corresponding author upon reasonable request.

## Supplementary Materials

The following are available online at https://www.mdpi.com/article/10.3390/cancers13112689/s1, Figure S1: Kaplan–Meier estimates of overall survival of patients with Galectin 9-positive tumors (high) vs. Galectin 9-negative/low-expressing tumors (low) assessed with immunochemistry on the TMA (left, *n* = 123, *p* = 0.31, log-rank test). Survival of patients from public transcriptomic databases with Galectin 9-low-expressing (≤66th percentile) vs. Galectin 9-high-expressing (>66th percentile) tumors. The overall survival is statistically equivalent (*n* = 510, *p* = 0.339, log-rank test, right). Figure S2: Overall survival related to the expression of the immune molecules. A: Kaplan–Meier estimates of overall survival of patients using a cutoff of 1. IDO, VISTA, and TIM3 high (scores 1 and 2) vs. IDO low (score 0) tumors assessed with immunochemistry on the TMA. The overall survival is statistically equivalent. B: Kaplan–Meier estimates of overall survival of patients with respect to the individual scores. The overall survival is statistically equivalent. The file ‘R script for public data analysis.txt’ containing the R script to produce the figures of the survival analysis of the publicly available transcriptomic data. The R script was modified from the original publication of Gendoo et al. [20].

## Abbreviations

FFPE	formalin-fixed, paraffin-embedded
DSF	disease-free survival
IDO	indoleamine 2,3-dioxygenase
LAG3	lymphocyte-activation gene 3
MHC II	major histocompatibility complex 2
OS	overall survival
PDAC	ductal adenocarcinoma of the pancreas
REMARK	reporting recommendations for tumor marker prognostic studies
TIL	tumor-infiltrating lymphocyte
TIM3	T cell immunoglobulin and mucin-domain containing-3
TMA	tissue microarrays
Treg	regulatory T cell
VISTA	V-domain Ig suppressor of T cell activation

## References

[1] Gordon-Dseagu V.L., Devesa S.S., Goggins M., Stolzenberg-Solomon R. (2018). Pancreatic cancer incidence trends: Evidence from the Surveillance, Epidemiology and End Results (SEER) population-based data. Int. J. Epidemiol..

[2] Weber J., Mandalà M., Del Vecchio M., Gogas H., Arance A.M., Cowey C.L., Dalle S., Schenker M., Chiarion-Sileni V., Marquez-Rodas I. (2017). Adjuvant Nivolumab versus Ipilimumab in Resected Stage III or IV Melanoma. N. Engl. J. Med..

[3] Forde P.M., Chaft J.E., Smith K.N., Anagnostou V., Cottrell T.R., Hellmann M.D., Zahurak M., Yang S.C., Jones D.R., Broderick S. (2018). Neoadjuvant PD-1 Blockade in Resectable Lung Cancer. N. Engl. J. Med..

[4] Schmid P., Adams S., Rugo H.S., Schneeweiss A., Barrios C.H., Iwata H., Diéras V., Hegg R., Im S.A., Shaw Wright G. (2018). Atezolizumab and Nab-Paclitaxel in Advanced Triple-Negative Breast Cancer. N. Engl. J. Med..

[5] Henriksen A., Dyhl-Polk A., Chen I., Nielsen D. (2019). Checkpoint inhibitors in pancreatic cancer. Cancer Treat. Rev..

[6] Abdkarimi S., Soofiyani S.R., Elham G., Abdolahi H.M., Safarzadeh E., Baradaran B. (2020). Targeting immune checkpoints: Building better therapeutic puzzle in pancreatic cancer combination therapy. Eur. J. Cancer Care.

[7] Christenson E.S., Jaffee E., Azad N.S. (2020). Current and emerging therapies for patients with advanced pancreatic ductal adenocarcinoma: A bright future. Lancet Oncol..

[8] Uyttenhove C., Pilotte L., Théate I., Stroobant V., Colau D., Parmentier N., Boon T., van den Eynde B.J. (2003). Evidence for a tumoral immune resistance mechanism based on tryptophan degradation by indoleamine 2,3-dioxygenase. Nat. Med..

[9] Ruffo E., Wu R.C., Bruno T.C., Workman C.J., Vignali D.A. (2019). Lymphocyte-activation gene 3 (LAG3): The next immune checkpoint receptor. Semin. Immunol..

[10] Mehta N., Maddineni S., Mathews I.I., Sperberg R.A.P., Huang P.-S., Cochran J.R. (2019). Structure and Functional Binding Epitope of V-domain Ig Suppressor of T Cell Activation. Cell Rep..

[11] Wang L., Rubinstein R., Lines J.L., Wasiuk A., Ahonen C., Guo Y., Lu L.-F., Gondek D., Wang Y., Fava R.A. (2011). VISTA, a novel mouse Ig superfamily ligand that negatively regulates T cell responses. J. Exp. Med..

[12] Wolf Y., Anderson A.C., Kuchroo V.K. (2019). TIM3 comes of age as an inhibitory receptor. Nat. Rev. Immunol..

[13] Zhu C., Anderson A.C., Schubart A., Xiong H., Imitola J., Khoury S., Zheng X.X., Strom T.B., Kuchroo V.K. (2005). The Tim-3 ligand galectin-9 negatively regulates T helper type 1 immunity. Nat. Immunol..

[14] Rangachari M., Zhu C., Sakuishi K., Xiao S., Karman J., Chen A., Angin M., Wakeham A., Greenfield E.A., Sobel R.A. (2012). Bat3 promotes T cell responses and autoimmunity by repressing Tim-3–mediated cell death and exhaustion. Nat. Med..

[15] Popp F.C., Popp M.C., Zhao Y., Betzler C., Kropf S., Garlipp B., Benckert C., Kalinski T., Lippert H., Bruns C.J. (2017). Protocol of the PANCALYZE trial: A multicenter, prospective study investigating the tumor biomarkers CXCR4, SMAD4, SOX9 and IFIT3 in patients with resected pancreatic adenocarcinoma to predict the pattern of recurrence of the disease. BMC Cancer.

[16] McShane L.M., Altman D.G., Sauerbrei W., Taube S.E., Gion M., Clark G.M., for the Statistics Subcommittee of the NCI-EORTC Working Group on Cancer Diagnostics (2005). REporting recommendations for tumour MARKer prognostic studies (REMARK). Br. J. Cancer.

[17] Loeser H., Kraemer M., Gebauer F., Bruns C., Schröder W., Zander T., Alakus H., Hoelscher A., Buettner R., Lohneis P. (2020). Indoleamine 2,3-Dioxygenase (IDO) Expression Is an Independent Prognostic Marker in Esophageal Adenocarcinoma. J. Immunol. Res..

[18] Loeser H., Kraemer M., Gebauer F., Bruns C., Schröder W., Zander T., Persa O.-D., Alakus H., Hoelscher A., Buettner R. (2019). The expression of the immune checkpoint regulator VISTA correlates with improved overall survival in pT1/2 tumor stages in esophageal adenocarcinoma. OncoImmunology.

[19] Gebauer F., Krämer M., Bruns C., Schlößer H.A., Thelen M., Lohneis P., Schröder W., Zander T., Alakus H., Buettner R. (2020). Lymphocyte activation gene-3 (LAG3) mRNA and protein expression on tumour infiltrating lymphocytes (TILs) in oesophageal adenocarcinoma. J. Cancer Res. Clin. Oncol..

[20] Gendoo D.M.A., Zon M., Sandhu V., Manem V.S.K., Ratanasirigulchai N., Chen G.M., Waldron L., Haibe-Kains B. (2019). MetaGxData: Clinically Annotated Breast, Ovarian and Pancreatic Cancer Datasets and their Use in Generating a Multi-Cancer Gene Signature. Sci. Rep..

[21] Huber W., Carey V.J., Gentleman R., Anders S., Carlson M., Carvalho B.S., Bravo H.C., Davis S., Gatto L., Girke T. (2015). Orchestrating high-throughput genomic analysis with Bioconductor. Nat. Methods.

[22] Schröder M.S., Culhane A.C., Quackenbush J., Haibe-Kains B. (2011). *survcomp*: An R/Bioconductor package for performance assessment and comparison of survival models. Bioinformatics.

[23] Dunkler D., Ploner M., Schemper M., Heinze G. (2018). Weighted Cox Regression Using the R Package coxphw. J. Stat. Softw..

[24] Schemper M., Wakounig S., Heinze G. (2009). The estimation of average hazard ratios by weighted Cox regression. Stat. Med..

[25] R Development Core Team (2008). R: A Language and Environment for Statistical Computing.

[26] RStudio Team (2015). RStudio: Integrated Development Environment for R.

[27] Lex A., Gehlenborg N. (2014). Sets and intersections. Nat. Methods.

[28] Bailey P., Initiative A.P.C.G., Chang D.K., Nones K., Johns A.L., Patch A.-M., Gingras M.-C., Miller D.K., Christ A.N., Bruxner T.J.C. (2016). Genomic analyses identify molecular subtypes of pancreatic cancer. Nature.

[29] Stromnes I.M., Hulbert A., Pierce R.H., Greenberg P.D., Hingorani S.R. (2017). T-cell Localization, Activation, and Clonal Expansion in Human Pancreatic Ductal Adenocarcinoma. Cancer Immunol. Res..

[30] Carstens J.L., De Sampaio P.C., Yang D., Barua S., Wang H., Rao A., Allison J.P., LeBleu V.S., Kalluri R. (2017). Spatial computation of intratumoral T cells correlates with survival of patients with pancreatic cancer. Nat. Commun..

[31] Balli D., Rech A.J., Stanger B.Z., Vonderheide R.H. (2017). Immune Cytolytic Activity Stratifies Molecular Subsets of Human Pancreatic Cancer. Clin. Cancer Res..

[32] Lohneis P., Sinn M., Bischoff S., Jühling A., Pelzer U., Wislocka L., Bahra M., Sinn B.V., Denkert C., Oettle H. (2017). Cytotoxic tumour-infiltrating T lymphocytes influence outcome in resected pancreatic ductal adenocarcinoma. Eur. J. Cancer.

[33] Robert C. (2020). A decade of immune-checkpoint inhibitors in cancer therapy. Nat. Commun..

[34] Royal R.E., Levy C., Turner K., Mathur A., Hughes M., Kammula U.S., Sherry R.M., Topalian S.L., Yang J.C., Lowy I. (2010). Phase 2 Trial of Single Agent Ipilimumab (Anti-CTLA-4) for Locally Advanced or Metastatic Pancreatic Adenocarcinoma. J. Immunother..

[35] Brahmer J.R., Tykodi S.S., Chow L.Q.M., Hwu W.-J., Topalian S.L., Hwu P., Drake C.G., Camacho L.H., Kauh J., Odunsi K. (2012). Safety and Activity of Anti–PD-L1 Antibody in Patients with Advanced Cancer. N. Engl. J. Med..

[36] Le D.T., Durham J.N., Smith K.N., Wang H., Bartlett B.R., Aulakh L.K., Lu S., Kemberling H., Wilt C., Luber B.S. (2017). Mismatch repair deficiency predicts response of solid tumors to PD-1 blockade. Science.

[37] O’Reilly E.M., Oh D.-Y., Dhani N., Renouf D.J., Lee M.A., Sun W., Fisher G., Hezel A., Chang S.-C., Vlahovic G. (2019). Durvalumab with or without Tremelimumab for Patients with Metastatic Pancreatic Ductal Adenocarcinoma. JAMA Oncol..

[38] Dranoff G., Jaffee E., Lazenby A., Golumbek P., Levitsky H., Brose K., Jackson V., Hamada H., Pardoll D., Mulligan R.C. (1993). Vaccination with irradiated tumor cells engineered to secrete murine granulocyte-macrophage colony-stimulating factor stimulates potent, specific, and long-lasting anti-tumor immunity. Proc. Natl. Acad. Sci. USA.

[39] Steele C.W., Karim S.A., Leach J.D., Bailey P., Upstill-Goddard R., Rishi L., Foth M., Bryson S., McDaid K., Wilson Z. (2016). CXCR2 Inhibition Profoundly Suppresses Metastases and Augments Immunotherapy in Pancreatic Ductal Adenocarcinoma. Cancer Cell.

[40] You F., Jiang L., Zhang B., Lu Q., Zhou Q., Liao X., Wu H., Du K., Zhu Y., Meng H. (2016). Phase 1 clinical trial demonstrated that MUC1 positive metastatic seminal vesicle cancer can be effectively eradicated by modified Anti-MUC1 chimeric antigen receptor transduced T cells. Sci. China Life Sci..

[41] Le D.T., Picozzi V.J., Ko A.H., Wainberg Z.A., Kindler H., Wang-Gillam A., Oberstein P.E., Morse M.A., Zeh H.J., Weekes C.D. (2019). Results from a Phase IIb, Randomized, Multicenter Study of GVAX Pancreas and CRS-207 Compared with Chemotherapy in Adults with Previously Treated Metastatic Pancreatic Adenocarcinoma (ECLIPSE Study). Clin. Cancer Res..

[42] Five Prime Therapeutics (2020). Five Prime Therapeutics Provides Update on Phase 2 Trial of Cabiralizumab Combined with Opdivo in Pancreatic Cancer [News Release].

[43] Upadhrasta S., Zheng L. (2019). Strategies in Developing Immunotherapy for Pancreatic Cancer: Recognizing and Correcting Multiple Immune “Defects” in the Tumor Microenvironment. J. Clin. Med..

[44] Chawla A., Molina G., Pak L.M., Rosenthal M., Mancias J.D., Clancy T.E., Wolpin B.M., Wang J. (2019). Neoadjuvant Therapy is Associated with Improved Survival in Borderline-Resectable Pancreatic Cancer. Ann. Surg. Oncol..

[45] Munn D.H., Sharma M.D., Hou D., Baban B., Lee J.R., Antonia S.J., Messina J.L., Chandler P., Koni P.A., Mellor A.L. (2004). Expression of indoleamine 2,3-dioxygenase by plasmacytoid dendritic cells in tumor-draining lymph nodes. J. Clin. Investig..

[46] Munn D.H., Sharma M.D., Baban B., Harding H.P., Zhang Y., Ron D., Mellor A.L. (2005). GCN2 Kinase in T Cells Mediates Proliferative Arrest and Anergy Induction in Response to Indoleamine 2,3-Dioxygenase. Immunity.

[47] Nishi M., Yoshikawa K., Higashijima J., Tokunaga T., Kashihara H., Takasu C., Ishikawa D., Wada Y., Shimada M. (2018). The Impact of Indoleamine 2,3-dioxygenase (IDO) Expression on Stage III Gastric Cancer. Anticancer Res..

[48] Muller A.J., DuHadaway J.B., Donover P.S., Sutanto-Ward E., Prendergast G.C. (2005). Inhibition of indoleamine 2,3-dioxygenase, an immunoregulatory target of the cancer suppression gene Bin1, potentiates cancer chemotherapy. Nat. Med..

[49] Patil P.A., Blakely A.M., Lombardo K.A., Machan J.T., Miner T.J., Wang L.-J., Marwaha A.S., Matoso A. (2018). Expression of PD-L1, indoleamine 2,3-dioxygenase and the immune microenvironment in gastric adenocarcinoma. Histopathology.

[50] Jacquemier J., Bertucci F., Finetti P., Esterni B., Charafe-Jauffret E., Thibult M.-L., Houvenaeghel G., Eynde B.V.D., Birnbaum D., Olive D. (2011). High expression of indoleamine 2,3-dioxygenase in the tumour is associated with medullary features and favourable outcome in basal-like breast carcinoma. Int. J. Cancer.

[51] Heeren M., Van Dijk I., Berry D.R.A.I., Khelil M., Ferns D., Kole J., Musters R.J.P., Thijssen V.L., Mom C.H., Kenter G.G. (2018). Indoleamine 2,3-Dioxygenase Expression Pattern in the Tumor Microenvironment Predicts Clinical Outcome in Early Stage Cervical Cancer. Front. Immunol..

[52] Riesenberg R., Weiler C., Spring O., Eder M., Buchner A., Popp T., Castro M., Kammerer R., Takikawa O., Hatz R.A. (2007). Expression of Indoleamine 2,3-Dioxygenase in Tumor Endothelial Cells Correlates with Long-term Survival of Patients with Renal Cell Carcinoma. Clin. Cancer Res..

[53] Sideras K., Biermann K., Yap K., Mancham S., Boor P.P., Hansen B.E., Stoop H.J., Peppelenbosch M., Van Eijck C.H., Sleijfer S. (2017). Tumor cell expression of immune inhibitory molecules and tumor-infiltrating lymphocyte count predict cancer-specific survival in pancreatic and ampullary cancer. Int. J. Cancer.

[54] Seifert L., Plesca I., Müller L., Sommer U., Heiduk M., von Renesse J., Digomann D., Glück J., Klimova A., Weitz J. (2021). LAG-3-Expressing Tumor-Infiltrating T Cells Are Associated with Reduced Disease-Free Survival in Pancreatic Cancer. Cancers.

[55] Leone P., Buonavoglia A., Fasano R., Solimando A.G., De Re V., Cicco S., Vacca A., Racanelli V. (2019). Insights into the Regulation of Tumor Angiogenesis by Micro-RNAs. J. Clin. Med..

