# Supplementary material for: Expression of Immune Checkpoint Regulators IDO, VISTA, LAG3, and TIM3 in Resected Pancreatic Ductal Adenocarcinoma
Source: Cancers (Basel). 2021 May 29;13(11):2689. doi: 10.3390/cancers13112689 (PMC8198722; doi:10.3390/cancers13112689)
Supplement: Supplementary file 1 [file cancers-13-02689-s001.zip › Figure S1-S2.pdf]

Expression of Immune Checkpoint Regulators IDO, VISTA, LAG3, and TIM3 in Resected Pancreatic Ductal Adenocarcinoma

Felix C. Popp, Ingracia Capino, Joana Bartels, Alexander I. Damanakis, Jiahui Li, Rabi R. Datta, Heike Löser, Yue Zhao, Alexander Quaas, Philipp Lohneis, Christiane J. Bruns and on behalf of the Pancalyze study Group

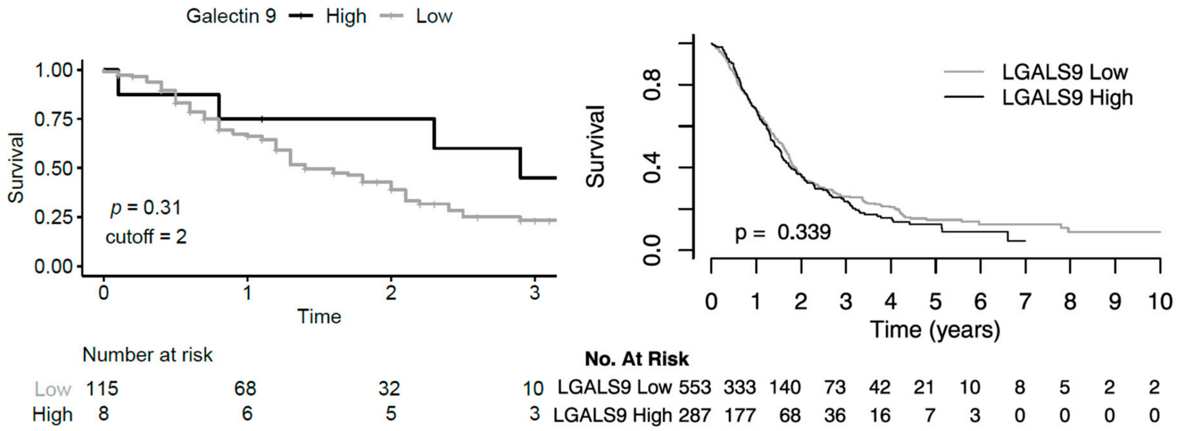

Figure S1. Kaplan–Meier estimates of overall survival of patients with Galectin 9-positive tumors (high) vs. Galectin 9-negative/low-expressing tumors (low) assessed with immunochemistry on the TMA (left, *n* = 123, *p* = 0.31, log-rank test). Survival of patients from public transcriptomic databases with Galectin 9-low-expressing (≤66th percentile) vs. Galectin 9-high-expressing (>66th percentile) tumors. The overall survival is statistically equivalent (*n* = 510, *p* = 0.339, log-rank test, right).

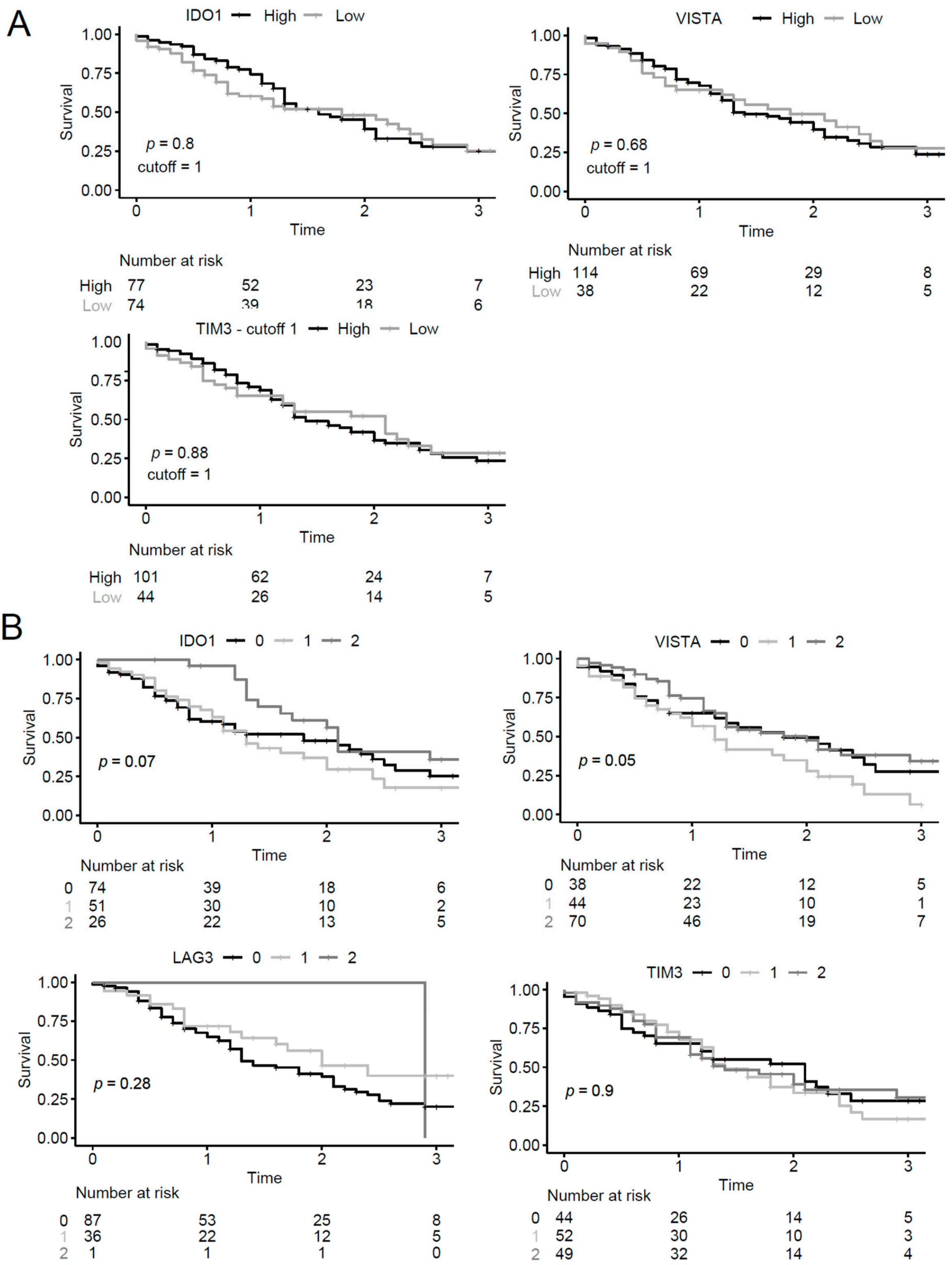

Figure S2. Overall survival related to the expression of the immune molecules. A: Kaplan–Meier estimates of overall survival of patients using a cutoff of 1. IDO, VISTA, and TIM3 high (scores 1 and 2) vs. IDO low (score 0) tumors assessed with immunochemistry on the TMA. The overall survival is statistically equivalent. B: Kaplan–Meier estimates of overall survival of patients with respect to the individual scores. The overall survival is statistically equivalent.
